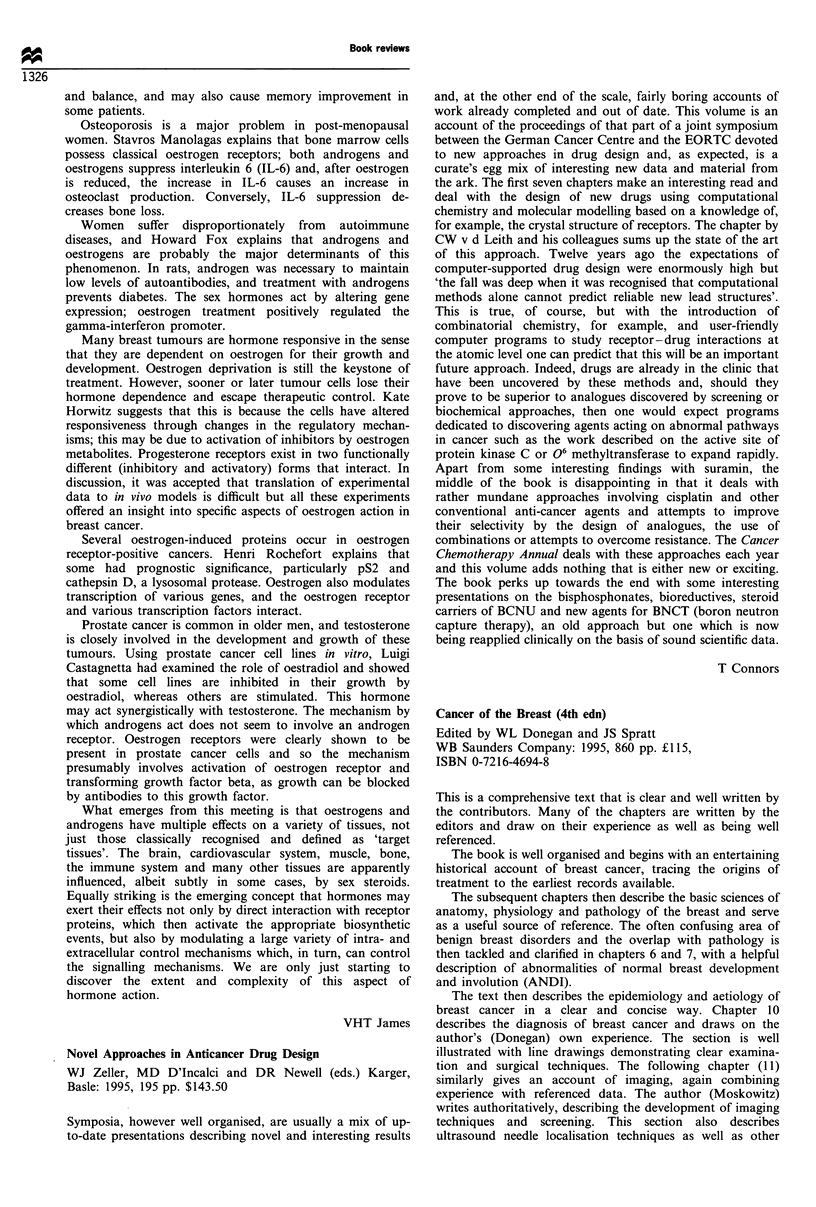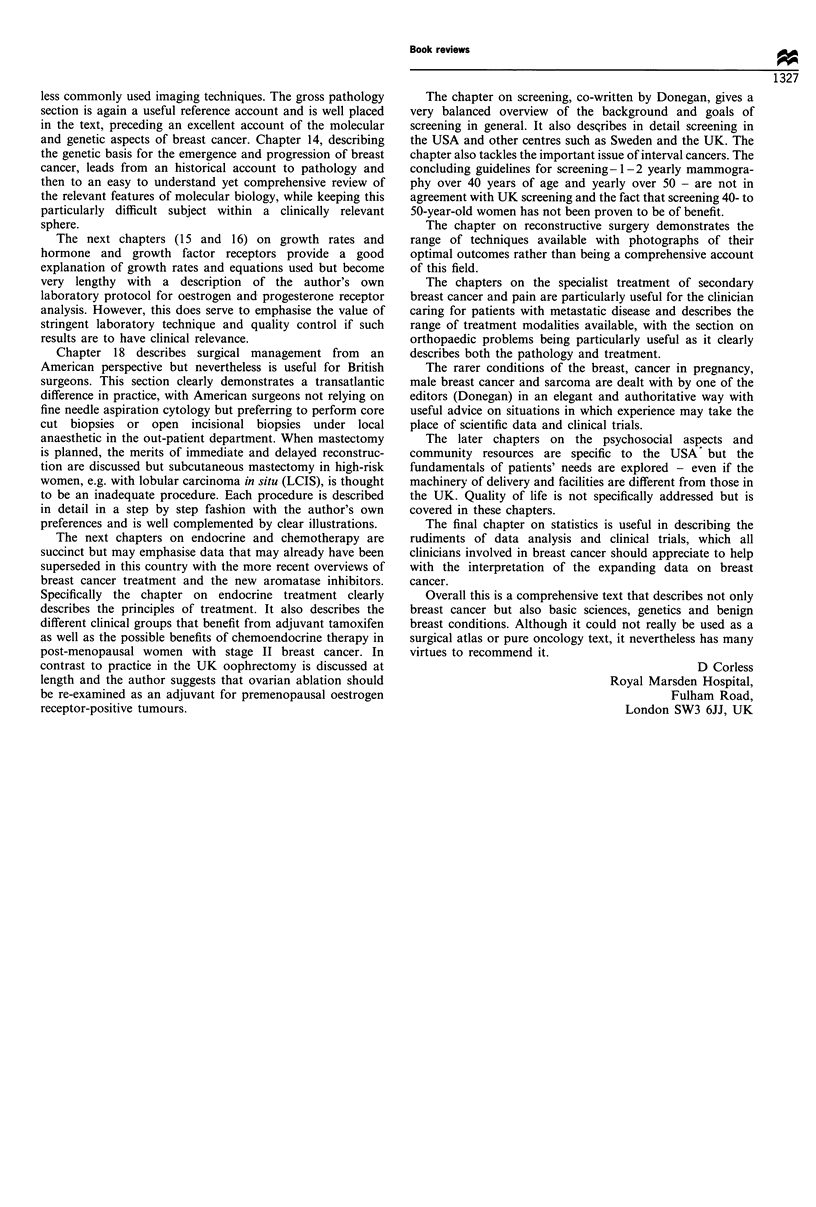# Cancer of the breast (4th edn)

**Published:** 1996-10

**Authors:** D Corless


**Cancer of the Breast (4th edn)**

Edited by WL Donegan and JS Spratt
WB Saunders Company: 1995, 860 pp. £115,
ISBN 0-7216-4694-8

This is a comprehensive text that is clear and well written by the contributors. Many of the chapters are written by the editors and draw on their experience as well as being well referenced.

The book is well organised and begins with an entertaining historical account of breast cancer, tracing the origins of treatment to the earliest records available.

The subsequent chapters then describe the basic sciences of anatomy, physiology and pathology of the breast and serve as a useful source of reference. The often confusing area of benign breast disorders and the overlap with pathology is then tackled and clarified in chapters 6 and 7, with a helpful description of abnormalities of normal breast development and involution (ANDI).

The text then describes the epidemiology and aetiology of breast cancer in a clear and concise way. Chapter 10 describes the diagnosis of breast cancer and draws on the author's (Donegan) own experience. The section is well illustrated with line drawings demonstrating clear examination and surgical techniques. The following chapter (11) similarly gives an account of imaging, again combining experience with referenced data. The author (Moskowitz) writes authoritatively, describing the development of imaging techniques and screening. This section also describes ultrasound needle localisation techniques as well as other less commonly used imaging techniques. The gross pathology section is again a useful reference account and is well placed in the text, preceding an excellent account of the molecular and genetic aspects of breast cancer. Chapter 14, describing the genetic basis for the emergence and progression of breast cancer, leads from an historical account to pathology and then to an easy to understand yet comprehensive review of the relevant features of molecular biology, while keeping this particularly difficult subject within a clinically relevant sphere.

The next chapters (15 and 16) on growth rates and hormone and growth factor receptors provide a good explanation of growth rates and equations used but become very lengthy with a description of the author's own laboratory protocol for oestrogen and progesterone receptor analysis. However, this does serve to emphasise the value of stringent laboratory technique and quality control if such results are to have clinical relevance.

Chapter 18 describes surgical management from an American perspective but nevertheless is useful for British surgeons. This section clearly demonstrates a transatlantic difference in practice, with American surgeons not relying on fine needle aspiration cytology but preferring to perform core cut biopsies or open incisional biopsies under local anaesthetic in the out-patient department. When mastectomy is planned, the merits of immediate and delayed reconstruction are discussed but subcutaneous mastectomy in high-risk women, e.g. with lobular carcinoma *in situ* (LCIS), is thought to be an inadequate procedure. Each procedure is described in detail in a step by step fashion with the author's own preferences and is well complemented by clear illustrations.

The next chapters on endocrine and chemotherapy are succinct but may emphasise data that may already have been superseded in this country with the more recent overviews of breast cancer treatment and the new aromatase inhibitors. Specifically the chapter on endocrine treatment clearly describes the principles of treatment. It also describes the different clinical groups that benefit from adjuvant tamoxifen as well as the possible benefits of chemoendocrine therapy in post-menopausal women with stage II breast cancer. In contrast to practice in the UK oophrectomy is discussed at length and the author suggests that ovarian ablation should be re-examined as an adjuvant for premenopausal oestrogen receptor-positive tumours.

The chapter on screening, co-written by Donegan, gives a very balanced overview of the background and goals of screening in general. It also describes in detail screening in the USA and other centres such as Sweden and the UK. The chapter also tackles the important issue of interval cancers. The concluding guidelines for screening – 1 – 2 yearly mammography over 40 years of age and yearly over 50 – are not in agreement with UK screening and the fact that screening 40- to 50-year-old women has not been proven to be of benefit.

The chapter on reconstructive surgery demonstrates the range of techniques available with photographs of their optimal outcomes rather than being a comprehensive account of this field.

The chapters on the specialist treatment of secondary breast cancer and pain are particularly useful for the clinician caring for patients with metastatic disease and describes the range of treatment modalities available, with the section on orthopaedic problems being particularly useful as it clearly describes both the pathology and treatment.

The rarer conditions of the breast, cancer in pregnancy, male breast cancer and sarcoma are dealt with by one of the editors (Donegan) in an elegant and authoritative way with useful advice on situations in which experience may take the place of scientific data and clinical trials.

The later chapters on the psychosocial aspects and community resources are specific to the USA but the fundamentals of patients' needs are explored – even if the machinery of delivery and facilities are different from those in the UK. Quality of life is not specifically addressed but is covered in these chapters.

The final chapter on statistics is useful in describing the rudiments of data analysis and clinical trials, which all clinicians involved in breast cancer should appreciate to help with the interpretation of the expanding data on breast cancer.

Overall this is a comprehensive text that describes not only breast cancer but also basic sciences, genetics and benign breast conditions. Although it could not really be used as a surgical atlas or pure oncology text, it nevertheless has many virtues to recommend it.

D Corless
Royal Marsden Hospital,
Fulham Road,
London SW3 6JJ, UK